# Case Report: Malignant transformation of maxillary giant cell tumor of bone from EURACAN reference center

**DOI:** 10.3389/fonc.2025.1604056

**Published:** 2025-07-17

**Authors:** Federica Riva, Sabrina Vari, Concetta Elisa Onesti, Renato Covello, Silvia Scuderi, Serena Ceddia, Maria Rosaria Fiore, Vincenzo Anelli, Sabino Strippoli, Virginia Ferraresi

**Affiliations:** ^1^ UOSD Sarcomas and Rare Tumors, IRCCS Regina Elena National Cancer Institute, Sapienza University of Rome, Rome, Italy; ^2^ UOSD Sarcomas and Rare Tumors, IRCCS Regina Elena National Cancer Institute, Rome, Italy; ^3^ Department of Pathology, IRCCS Regina Elena National Cancer Institute, Rome, Italy; ^4^ Department of Radiological, Oncological and Pathological Sciences, Sapienza, University of Rome, Rome, Italy; ^5^ Radiation Oncology Unit, Clinical Department, CNAO National Center for Oncological Hadrontherapy, Pavia, Italy; ^6^ Radiology Department, IRCCS Regina Elena National Cancer Institute, Rome, Italy; ^7^ Rare Tumors and Melanoma Unit, Istituto di Ricovero e Cura a Carattere Scientifico (IRCCS) Istituto Tumori Giovanni Paolo II, Bari, Italy

**Keywords:** giant cell tumor of bone, sarcoma, malignant transformation, hadrontherapy, case report

## Abstract

Giant cell tumor of bone (GCTB) is a benign but locally aggressive neoplasm that can rarely undergo malignant transformation, with a poor prognosis. The most frequent histotypes of the sarcomatous transformation of GCTB are osteosarcoma, fibrosarcoma, and undifferentiated pleomorphic sarcoma, and the treatment approach mirrors that of high-grade sarcomas. This case report describes the malignant transformation of a maxillary GCTB treated with standard systemic treatments for bone tumors and local treatment, resulting in a progression of disease until the patient’s death. Nevertheless, a marked radiological and clinical response was achieved with local carbon ion therapy. This case highlights the diagnostic and therapeutic challenges of malignant transformation of GCTB, emphasizing the importance of a multidisciplinary approach at specialized centers and the potential role of local therapies in selected cases.

## Introduction

Giant cell tumor of bone (GCTB) is an uncommon, benign tumor typically affecting the epiphyseal regions of long bones, such as the distal femur and proximal tibia. The primary treatment for GCTB is surgical resection, which is often curative (1). To date, the only medical treatment approved for unresectable GCTB is denosumab (1).

Although GCTB is generally considered a locally aggressive benign tumor, its potential to metastasize is not unusual, with lung metastases occurring in 1%–9% of cases (1).

Even more rarely, GCTB can undergo sarcomatous transformation associated with more aggressive behavior and a poor prognosis, with an increased risk of metastasis and recurrence. Malignant GCTBs are comparable to a high-grade sarcoma, such as undifferentiated sarcoma or osteosarcoma, and treatment may accordingly include peri-operative chemotherapy and/or radiation therapy in addition to surgery (2–6). No specific guidelines on the medical approach to these rare neoplasms of bone are currently available, and osteosarcoma-like chemotherapy regimens are generally used. Although the underlying mechanisms of malignant transformation remain incompletely understood, genetic, epigenetic, and microenvironmental factors appear to play crucial roles (7, 8). Despite the recent adoption of H3F3A immunohistochemistry, which is commonly positive in GCTB, the H3F3A mutation can sometimes be lost when the tumor undergoes malignant transformation, providing an important diagnostic approach (7, 8).

Despite this, the malignant transformation of GCTB presents unique challenges in both diagnosis and treatment. Here, we present a case of malignant transformation of maxillary GCTB to explore the management, treatment, and clinical implications. The case report was written according to the CARE, European Society for Medical Oncology (ESMO), and European Network for Rare adult solid Cancer (EURACAN) guidelines.

## Case description

In 2013, a 20-year-old man underwent local excision of a maxillary mass, with a histopathological diagnosis of fibrous dysplasia. The patient’s medical history was negative for McCune–Albright syndrome.

From 2020 to 2022, for multiple local recurrences of maxillary lesions, the patient underwent several local excisions and a right hemimaxillectomy, which included the resection of the hard palate and palatal mucosa with reconstruction using an osteomuscular flap from the right iliac crest, with histopathological confirmation of fibrous dysplasia.

In August 2022, a CT scan confirmed further local recurrence with a lesion of 30 × 25 mm on the hard palate, responsible for the erosion of the bone flap.

After a biopsy with a histological diagnosis of GCTB, the entire lesion was surgically removed with histopathological confirmation of the histotype.

In January 2023, an MRI showed the appearance of a relapse of disease with a new lesion originating in the right maxillary sinus and a second lesion in the ipsilateral nasolacrimal duct. A total body CT scan excluded distant metastases.

Considering the local extension of these neoformations, no indication was given for loco-regional surgical treatment.

From February 2023 to May 2023, the patient underwent treatment with denosumab 120 mg q28 days after three weekly administrations as loading dose, with a significant volumetric increase in the pathological solid tissue associated with an invasion and replacement of the right maxillary sinus and the right orbital cavity, also extensively affecting the right nasal cavity and the hard palate, as documented by CT and MRI (9, 10).

In June 2023, the patient was referred to our institute, which is a EURACAN referral center on sarcomas. At the moment of our evaluation, the patient presented with a large mass protruding from the oral cavity with alteration of the facial mass, with associated right hearing loss, right alteration of vision, and difficulty breathing due to obstruction of the oral cavity. After a tracheotomy procedure and subsequent percutaneous gastrostomy, it was decided to perform a further biopsy of the hard palate lesion and a histological review of the previous surgery.

Both histopathological examinations were suggestive of a malignant transformation of GCTB, like an undifferentiated sarcoma.

**Figure 1:**
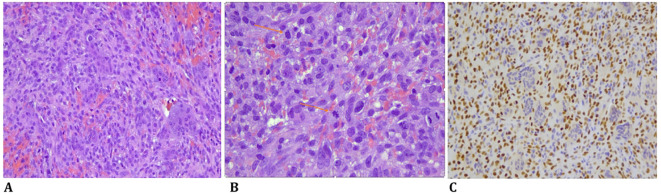
**(A)** Spindle cell high-grade sarcoma with numerous mitotic figures, some of which are atypical, and scattered osteoclast-like giant cells (HE, original magnification, ×200). **(B)** A high-grade sarcoma with numerous atypical mitoses and scattered osteoclast-like giant cells (HE, original magnification, ×200). **(C)** Immunohistochemistry for SATB2 was positive in the malignant cells (SATB2 immunostaining, original magnification, ×200).

The biopsy material showed a proliferation of atypical spindle cells with open chromatin and evident large nucleoli. Numerous mitotic figures were found, some of which were atypical. Scattered between the malignant cells, there were also some osteoclast-like giant cells (**Figures 1A, B**).

Immunohistochemically, the atypical spindle cells were negative for H3F3A and positive for SATB2 (**Figure 1C**) (8, 11, 12). From July to August 2023, the patient was treated with two cycles of chemotherapy, including Adriamycin and cisplatin, according to the EUROBOSS scheme, resulting in a moderate radiological reduction in tumor volume, as shown in the MRI in September 2023 (13).

In the absence of surgical indications, the patient continued the chemotherapy treatment as a good responder according to the EUROBOSS scheme, but after only one cycle of Adriamycin, the patient presented a local clinical progression of the disease with epistaxis and bleeding from the oral cavity.

**Figure 2:**
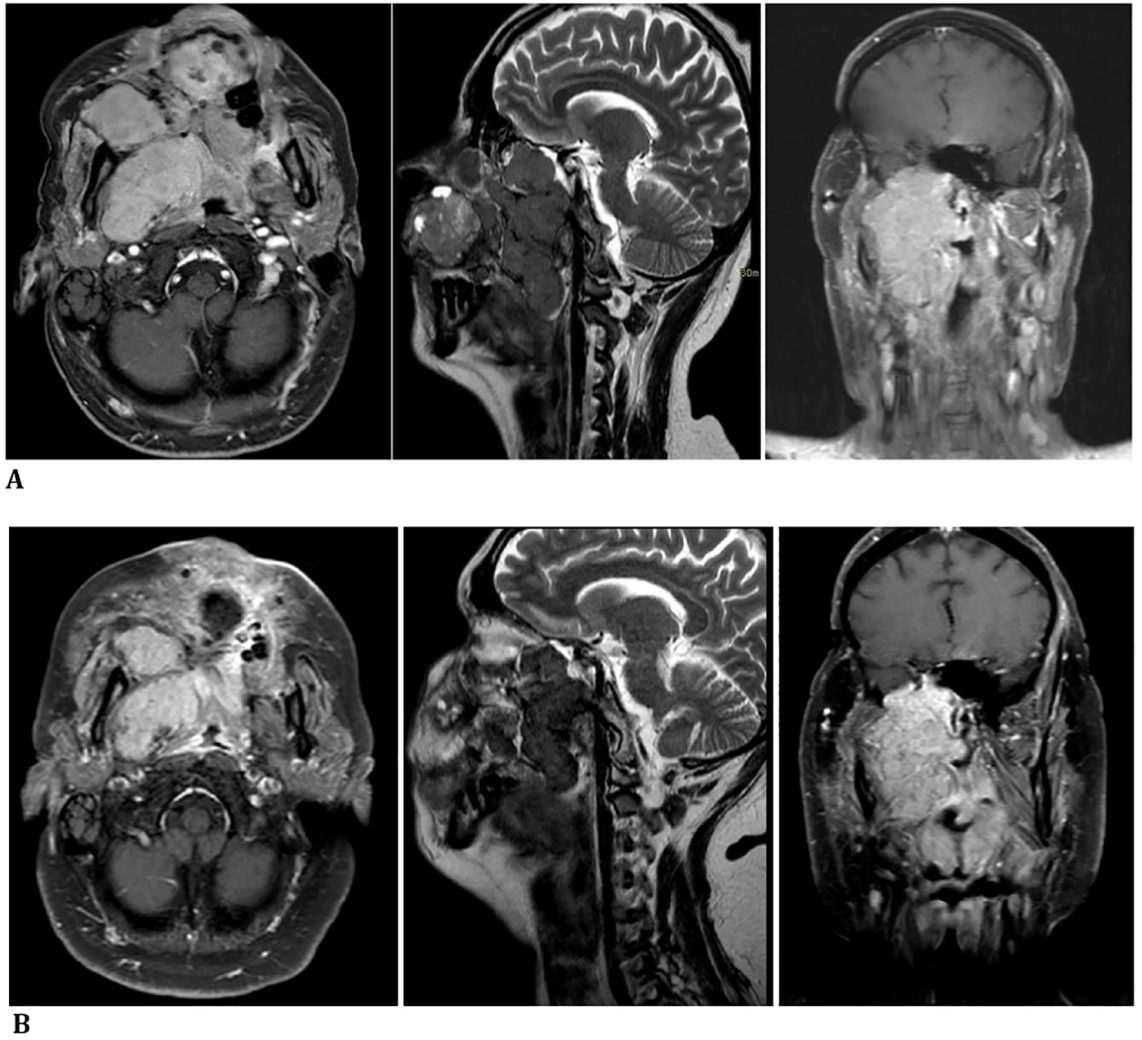
**(A)** October 2023—progression of disease confirmed by a facial massif MRI before carbon ion therapy. **(B)** December 2023—radiological reduction in tumor size shown in a facial massif MRI after carbon ion therapy.

A facial MRI confirmed radiological evidence of the progression of disease (**Figure 2A**).

In view of progression during chemotherapy and highly disabling symptoms related to local progression in the absence of distant disease, hadrontherapy was proposed as a targeted treatment strategy to optimize local control through the use of high-LET particles, ensuring superior dose precision and radiobiological efficacy (14–19).

From November to December 2023, at CNAO in Pavia, Italy, carbon ion therapy was administered to target the right maxillary sinus, nasal cavity, and hard palate with a total dose of 60 Gy in 20 fractions using the IMPT technique.

Local treatment showed clinical benefit and radiological reduction in tumor size, with the improvement of symptoms during and after therapy (**Figure 2B**).

In February 2024, with the intent to maintain the response to hadrontherapy, the patient was started on second-line treatment with ifosfamide 15 g/m^2^. Three cycles, with a 75% reduction from the second cycle for severe bone marrow toxicity with associated pneumonia and acute renal failure (Cr = 2.8) requiring hospitalization, were administered until May 2024 (20).

**Figure 3:**
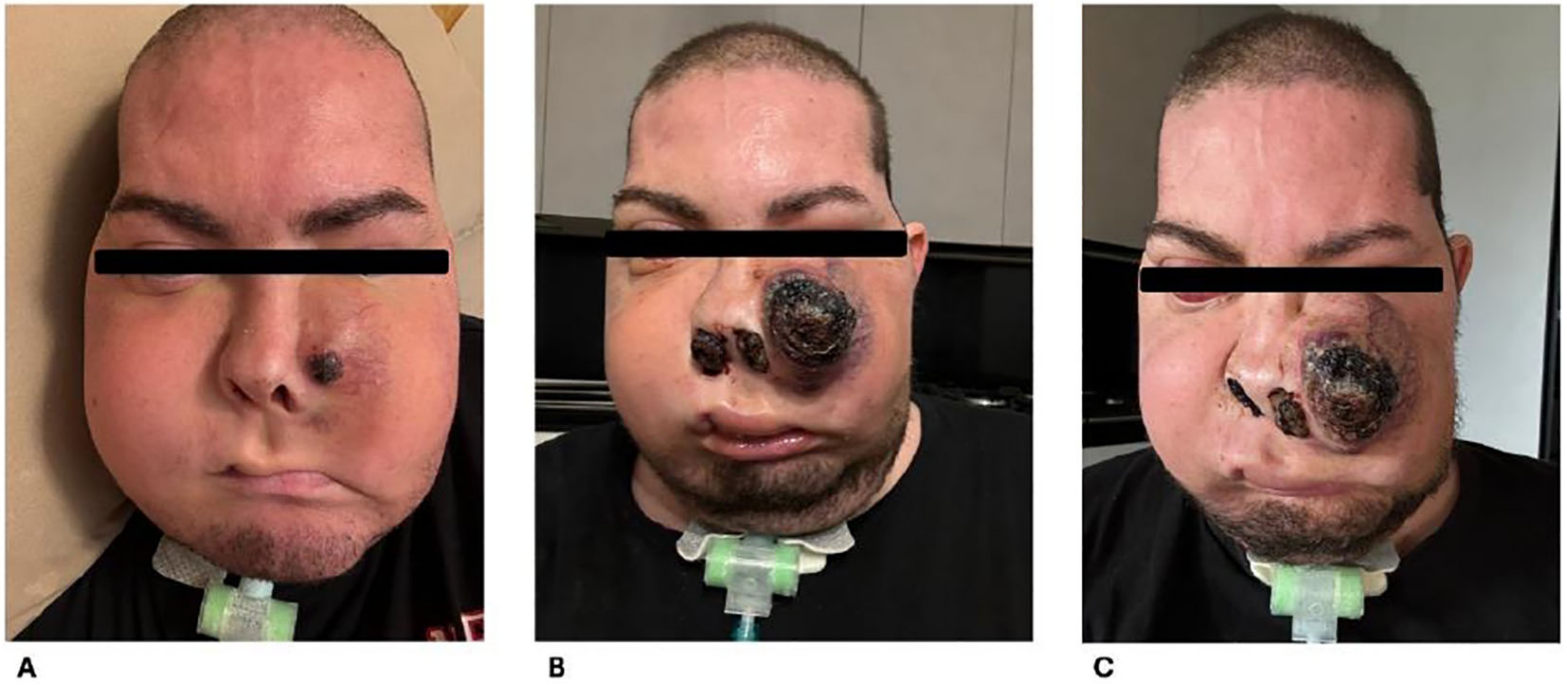
Clinical evolution of disease. **(A)** July 2024, increase in jaw swelling. **(B, C)** August 2024, clinical progression and appearance of ulcerated lesion in zygomatic area.

The MRI performed for the local restaging of disease documented an increase in the size of the neoformation above all on the level of the space in front of the ascending branch of the right mandible in the anterior maxillary region of the obliterated oral cavity; there was extension of the neoplastic tissue in the intraconic endorbital site on the right at the level of the floor and the mesial wall responsible for the imprint on the rectus muscles and newly appeared extra-axial endocranial extension of the neoformation with nodular pachymeningeal infiltration at the level of the polar portion of the right middle cranial fossa. Clinically, the patient presented with a noticeable increase in jaw swelling. Since June 2024, the patient received a new line of treatment with regorafenib 160 mg/day, of which he exhibited at only 1 month evidence of clinical progression and appearance of an ulcerated lesion in the left zygomatic area (21) (**Figure 3**).

**Figure 4:**
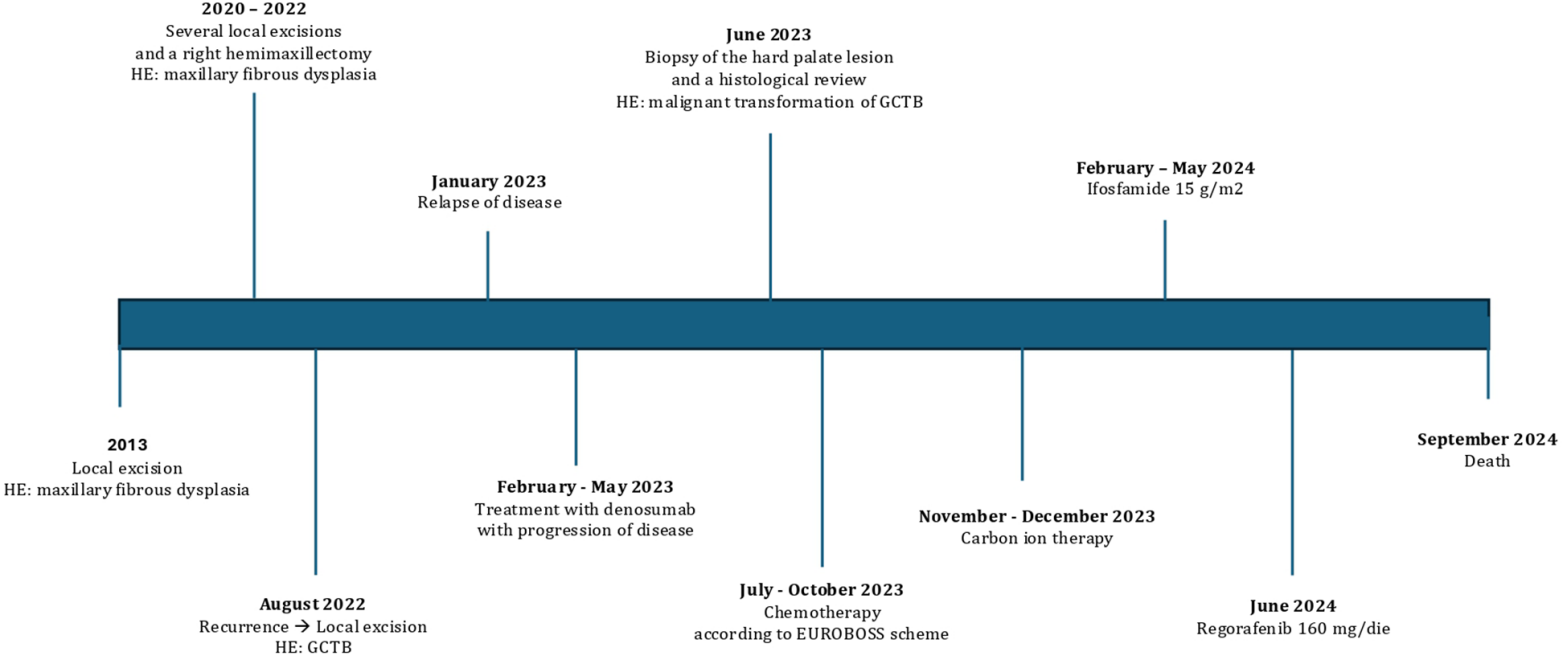
Clinical timeline from diagnosis to death. HE, histopathological examination; GCTB, giant cell tumor of bone.

Unfortunately, the disease was rapidly progressing, and the patient passed away in September 2024, with an Overall survival (OS) of 25 months from GCTB diagnosis, an OS of 15 months from the diagnosis of malignant transformation, and a time to the malignant transformation of 10 months (**Figure 4**).

## Discussion

The management of malignant GCTB remains challenging due to the rarity of the condition, and the prompt diagnosis of malignant transformation plays a crucial role in the early management of this aggressive disease. In the presented clinical case, the diagnosis of malignant transformation was confirmed after multiple local recurrences of GCTB treated surgically and after 4 months of treatment with denosumab. Previously, several studies have questioned whether denosumab may contribute to the malignant transformation of GCTB, with its activity on the immune system and on the process of inflammation and its inhibitory activity on RANKL (1). Till now, no study has definitively confirmed this correlation. The most plausible explanation appears to be an initial misdiagnosis rather than a direct association with the drug itself (22, 23). Therefore, to date, denosumab remains the only approved medical treatment for GCTB (1). A recent systematic review with meta-analysis confirmed the benefit both clinically and radiologically of denosumab regimens in the treatment of GCTB (24). In advanced GCTB, denosumab is used in a pre-operative setting to facilitate subchondral bone integrity and joint preservation surgery. To date, only Zheng has presented positive results of an adjuvant treatment with combined microwave ablation and curettage after neoadjuvant treatment with denosumab (25). Recently, the biological findings of the disease have been examined for the potential prognostic and therapeutic roles of some biomarkers and any targeted strategies. Recent studies have demonstrated the effectiveness of new-generation antiangiogenic drugs, such as lenvatinib, alone or in combination, in the management of this rare histotype. De Vita was the first to use patient-derived primary cultures with 2D and 3D culture platforms, confirming the efficacy of denosumab and demonstrating the promising role of lenvatinib in association with denosumab in the treatment of GCTB (26). A pilot study of lenvatinib plus pembrolizumab demonstrated a benefit in patients with advanced osteosarcoma, malignant peripheral nerve sheath tumor (MPNST), angiosarcoma, and synovial sarcoma (NCT04784247). Further studies are needed to better define the efficacy of these new drugs, already used in the treatment of many solid tumors (27). In the rare instances of sarcomatous transformation of GCTB, the most frequent histotypes are osteosarcoma, fibrosarcoma, or undifferentiated pleomorphic sarcoma, which lose H3F3A expression (8, 11, 12, 28). The treatment approach is generally the same for high-grade sarcomas, even though specific guidelines are lacking due to the rarity of the disease. In the case presented, after the diagnosis of malignant GCTB, the patient was initiated on chemotherapy according to the EUROBOSS protocol, which is commonly used for bone tumors (13). This treatment resulted in an initial radiological response but was rapidly followed by disease progression, both clinically and radiologically. In contrast to systemic therapy, a marked radiological response was achieved through local carbon ion radiotherapy (CIRT), which also provided clinical benefits during and after treatment. CIRT has demonstrated significant potential in treating radioresistant tumors due to its intrinsic physical selectivity, delivering high doses directly to the tumor while minimizing exposure to nearby critical tissues, even those in close proximity (14–17). Additionally, its superior radiobiological effectiveness is theoretically more common in radioresistant tumors (18). In this specific case, given the evidence of clinical disease behavior similar to high-grade osteosarcoma, CIRT was employed as the treatment approach. Subsequent standard systemic treatments for bone tumors proved ineffective, leading to disease progression and a gradual deterioration in the patient’s clinical condition, culminating in his death. This scenario suggests that CIRT may offer potential benefits, especially in particular cases of radioresistant tumors where the disease sites are difficult to treat surgically. Conventional radiotherapy often fails to achieve local control in cases of unresectable tumors or macroscopic residual disease. Controlling the growth of radioresistant tumors necessitates high doses of radiation; however, the ability to deliver such doses is frequently constrained by the proximity of critical structures within the targeted area (20). The anatomical site has an important impact on the outcomes, influencing both the feasibility of aggressive surgical approaches, aimed at complete resection, and the capacity to administer high-dose radiotherapy. However, more lines of evidence are needed to determine the efficacy and safety of CIRT. Further similar cases would be needed to assess and compare the clinical and radiological outcomes of radiotherapy versus systemic treatment. Nevertheless, reference centers are fundamental in the management of rare tumors, and the multidisciplinary approach remains a mainstay for the treatment of the disease and the management of the well-being of the patient.

## Conclusions

The case presented reinforces the poor prognosis and typical aggressiveness of the sarcomatous transformation of GCTB (4–6). Given the limited number of cases reported in the literature, a precise diagnostic–therapeutic framework is yet to be established. Nonetheless, an early and accurate diagnosis of malignant transformation remains essential for the correct therapeutic management. In this context, the case highlights the importance of histological evaluation in specialized referral centers with expertise in rare pathologies.

## Data Availability

The original contributions presented in the study are included in the article/supplementary material. Further inquiries can be directed to the corresponding author.
